# Spectral Entropic Radiomics Feature Extraction (SERFE): an adaptive approach for glioblastoma disease classification

**DOI:** 10.3389/frai.2025.1583079

**Published:** 2025-07-16

**Authors:** V. L. Sowmya, A. Bharathi Malakreddy, Santhi Natarajan, N. Prathik, I. S. Rajesh

**Affiliations:** ^1^Department of Artificial Intelligence & Machine Learning, BMS Institute of Technology & Management, Bangalore, India; ^2^Department of CSE, Shiv Nadar University, Chennai, India; ^3^Itron Inc., Bangalore, India

**Keywords:** spectral radiomics, entropy-weighted feature selection, graph-theoretic encoding, adaptive radiomics modelling, glioblastoma classification, TCIA-based radiomics, feature redundancy reduction

## Abstract

**Introduction:**

Radiomics-based glioblastoma classification demands feature extraction techniques that can effectively capture tumor heterogeneity while maintaining computational efficiency. Conventional tools such as PyRadiomics and CaPTk rely on extensive handcrafted feature sets, which often result in redundancy and necessitate further optimization steps.

**Methods:**

This study proposes a novel framework, Spectral Entropic Radiomics Feature Extraction (SERFE), which integrates spectral frequency decomposition, entropy-driven feature selection, and graph-based spatial encoding. SERFE decomposes voxel intensity fluctuations into spectral signatures, employs entropy-based weighting to prioritize informative features, and preserves spatial topology through graph-based modeling. The method was evaluated using the public TCIA glioblastoma dataset.

**Results:**

SERFE generated a refined feature set of 350 radiomic features from an initial pool of 2,260, achieving a 92% stability score and 91.7% classification accuracy. This performance surpasses traditional radiomics methods in both predictive accuracy and feature compactness.

**Discussion:**

The results demonstrate SERFE’s capacity to enhance tumor characterization and streamline radiomics pipelines without requiring post-extraction feature reduction. Its compatibility with existing clinical workflows makes it a promising tool for future neuro-oncology applications.

## 1 Introduction

Glioblastoma Multiforme (GBM) is considered to be one of the lethal brain cancers, as it is highly proliferative, its recurrence rate is high, and it typically does not adequately respond to standard treatments. Patient survival rates are still extremely low, reaching post-diagnosis only a few months, and have not greatly improved, even as surgical techniques, radiation regimens, and chemotherapy regimens have all improved. These ongoing challenges in the clinical context have inspired efforts to pursue diagnostic and prognostic approaches that can accommodate the complex biology of GBM. In recent years, there has been a growing focus on non-invasive imaging biomarkers as potential tools to enhance tumor evaluation. Isolated efforts with conventional radiologic approaches, however, have not necessarily captured the broader dynamic range of intratumoral heterogeneity, particularly when used in isolation as a modality for treatment planning or prognostic estimate (Zhang et al., 2023). To address this need, radiomics has evolved as a computational approach to the extraction of quantifiable patterns from medical images to characterize tumor-specific features (such as intensity, spatial heterogeneity, texture, and shape).

In neurooncological applications, radiomics have been used to enhance tumor grading, outcome prediction, and individualized therapy stratification. The workflows are usually built upon multistep flows that perform image preprocessing, systematic feature extraction, dimensionality treatment, and classification. In this study, we introduce a new feature extraction method that is tailored to handle the imaging challenges commonly observed in glioblastoma (Zhou M. et al., 2021).

### 1.1 The radiomics pipeline for glioblastoma analysis

**Figure 1:**
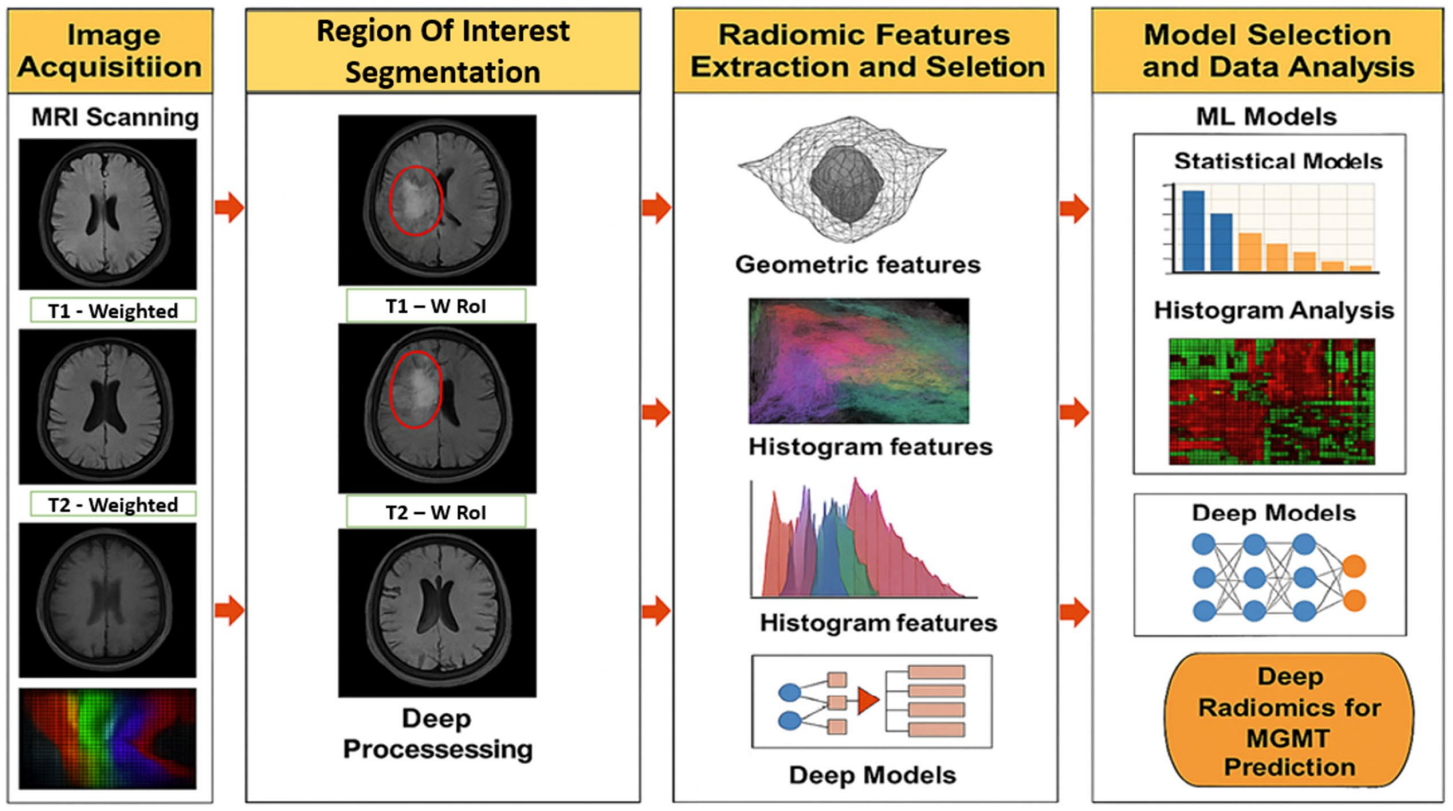
Radiomics pipeline.

Radiomics analysis is usually done using a structured pipeline that turns medical images into useful, measurable data that can be used in machine learning models. Figure 1 shows the steps in this pipeline, starting with getting the raw images and going through preprocessing, segmentation, and feature extraction, and finally ending with predictive modelling. To reduce variability between scanners, initial imaging usually includes T1-weighted, T2-weighted, FLAIR, or contrast-enhanced MRI scans. To make features more reliable, preprocessing steps like bias correction, normalization, and artifact removal are used. Then, either by hand or with automated algorithms, the tumor regions are divided into smaller parts called regions of interest (ROIs) from which features are taken (Napolitano and Gevaert, 2021).

Radiomics features can range from basic measures such as mean intensity to more complex descriptors that capture texture patterns and geometric shape information. These features are sensitive to tumor heterogeneity and are commonly used in classification or regression models to support clinical tasks such as identifying tumor grade, estimating patient prognosis, or anticipating treatment outcomes. More recently, radiomics has also contributed to studies on radiogenomic associations, offering insights that support personalized treatment planning tailored to individual patient profiles (Napolitano and Gevaert, 2021).

#### 1.1.1 Image acquisition and pre-processing

Radiomics analysis begins with the acquisition of medical images, most commonly through magnetic resonance imaging or computed tomography, which provide high-resolution anatomical data. In glioblastoma research, multi-parametric MRI sequences including T1-weighted, T2-weighted, contrast-enhanced T1 (T1-CE), and fluid-attenuated inversion recovery (FLAIR) are typically employed, as they capture complementary information on tumor morphology and internal heterogeneity. Large, annotated datasets are often accessed from public repositories such as The Cancer Imaging Archive (TCIA), supporting reproducibility and enabling multi-centre validation (Zhang et al., 2023).

Before features can be extracted, the images are preprocessed to address variability introduced by different scanners and imaging protocols. This ensures that extracted features reflect actual biological differences rather than technical artifacts. Standard preprocessing procedures involve bias field correction for intensity uniformity, normalization to standardize pixel value ranges, and artifact removal to suppress irrelevant signals (Zhou M. et al., 2021). Omitting these steps may lead to inconsistent feature sets and reduce the reliability of downstream predictive models.

#### 1.1.2 Tumor segmentation

Tumor segmentation is a foundational step in radiomics, as it defines the Region of Interest (ROI) from which features are derived. The accuracy of this step directly impacts the quality and relevance of the extracted features. Traditionally, manual segmentation performed by radiologists has been the gold standard; however, it is time-consuming and prone to inter-observer variability (Zwanenburg et al., 2020).

To overcome these limitations, many studies now employ automated or semi-automated segmentation techniques. In particular, deep learning-based architectures such as U-Net and its variants have gained traction due to their ability to learn complex spatial patterns and adapt across imaging datasets. These models have shown superior performance in segmenting tumor boundaries with high accuracy. Ensuring precise segmentation helps isolate tumor tissue from surrounding structures, minimizing feature contamination and enhancing the overall performance of classification models (Napolitano and Gevaert, 2021).

#### 1.1.3 Feature extraction

Once the tumor is delineated, radiomics attributes are gathered to represent its various characteristics. These attributes are classified into multiple categories:

- *First-order statistical features* describe intensity-based attributes, such as mean, variance, skewness, and kurtosis.
- *Texture-based features,* derived from Gray-Level Co-occurrence and Run Length Matrices capture spatial intensity relationships and heterogeneity patterns.
- *Shape-based features* define morphological properties such as sphericity, elongation, and compactness, providing insights into tumor geometry.
- *Higher-order features,* extracted using wavelet transformations and frequency-based decomposition, enhance discriminative power by revealing hidden tumor patterns.

The extracted features serve as the foundation for radiomics-based machine learning models. However, the high dimensionality of these feature sets often results in redundant and irrelevant data, necessitating further refinement (Napolitano and Gevaert, 2021).

#### 1.1.4 Feature selection and optimization

Not all extracted features contribute meaningfully to classification. Redundant and non-informative features introduce noise and increase computational complexity. To optimize feature selection, various statistical and machine learning-based techniques are employed:

- *Entropy-based selection* prioritizes features that maximize information gain.
- *Recursive Feature Elimination (RFE)* iteratively removes less significant features to retain the most relevant subset.
- *Principal Component Analysis (PCA)* reduces dimensions while maintaining variance.

Efficient feature selection ensures that only the most discriminative radiomics features are used, improving classification accuracy and computational efficiency.

#### 1.1.5 Classification and model training

The optimized feature set is used for glioblastoma classification, distinguishing tumor subtypes or non-tumor regions. Support Vector Machines and Random Forests are preferred due to their robustness in handling high-dimensional data with limited samples, making them ideal for biomedical applications (Zinn et al., 2021).

Machine learning models analyse radiomics and genomics data to improve diagnostic accuracy. Validation techniques like cross-validation ensure model reliability and prevent overfitting. A systematic evaluation of classifiers helps refine computational approaches for GBM diagnosis and subtype characterization.

### 1.2 Background work

#### 1.2.1 Importance of radiomics feature extraction in glioblastoma analysis

Feature extraction is an important step in radiomics that connects raw imaging data to computer analysis. This process allows for extracting tumor-specific features that might not be obvious from simply looking at the images in a form of structured numerical representations. These characteristics, such as shape, intensity distribution, texture deviations, and spatial configuration, form a high-dimensional data set that can be processed with algorithms to classify glioblastoma and related diseases.

The accuracy of radiomics-based diagnostic and prognostic tools largely depends on the quality of the features extracted. Selecting redundant or irrelevant features can introduce noise into models and compromise their generalization accuracy, particularly in high-dimensional datasets. For this reason, one of the priority aims is neuro-oncological image processing to develop feature extraction methods that are accurate and informative. Various methods for radiomics extraction have been developed in the past decade, each designed to obtain different tumor-related information. Three types of such strategies are:

- Hand-engineered Feature Extraction: PyRadiomics and CaPTk are representatives of traditional approaches to extract features based on predetermined algorithms for pixel intensity, shape features, and texture features (e.g., GLCM, GLRLM). Although such methods are understandable, they fail to account for complex spatial interactions.
- Wavelet-Based and Higher-Order Techniques: Methods that decompose images into multiple frequency bands have an option to analyse images at different resolutions. Features derived from such decompositions allow us to comprehend how texture varies across scales. This makes them particularly adept at sniffing out the differences in tumors.
- Deep Learning for Feature Extraction: Convolutional Neural Networks (CNNs) automatically learn a hierarchy of features from imaging data. Models of this kind have been effective at learning high-level representations and identities without the need for manual-engineering of features, but are typically dependent on large labelled data sets (Parekh and Jacobs, 2021). Each method has its own advantages and limitations depending on the dataset, additional preprocessing steps applied, and desired approach for clinical application. The comparison of such strategies is crucial in the selection of the best pipeline to classify GBM and predict its outcome.

#### 1.2.2 Existing radiomics feature extraction methods

This section provides a critical analysis of widely adopted radiomics feature extraction techniques used in glioblastoma classification. The comparison is based on fundamental aspects of feature extraction approach, computational efficiency, redundancy handling, and practicality in a clinical setting. The chosen methods include handcrafted and wavelet based as well as those stemming from deep learning to capture a range of complexity and performance (Zhang et al., 2023).

As outlined in Table 1, PyRadiomics and CaPTk extract pre-engineered features including shape, intensity, and texture descriptors. These methods, although well-established and interpretable, often produce large, high-dimensional feature sets. This increases the risk of redundancy and model overfitting, especially when applied to limited datasets. Post-processing techniques such as LASSO and mRMR are commonly employed to reduce feature overlap; however, these operate after extraction and may discard important features, limiting biological interpretability.

**Table 1:** Comparative analysis of radiomics feature extraction methods.

Feature extraction method	Feature extraction approach	Feature types extracted	Number of features extracted per patient	Computational complexity (Big-O notation)	Time per image (approx.)	Redundancy reduction	Clinical applicability
PyRadiomics	Statistical & texture-based analysis (GLCM, GLSZM, NGTDM)	First-order, texture, shape, wavelet-based	1,500 – 2,500	O (n × m) (*n* = features, m = tumor region)	~1.5 min per slice	Low (high correlation among extracted features)	Standardized but lacks adaptability
CaPTk	Predefined Feature Set	First-order, shape-based	500–1,000	O(n) (fixed feature set)	~2 min per image	Low (fixed feature set limits adaptability)	Reproducible, clinically validated but non-adaptive
Wavelet-Based Radiomics	Multi-Scale Frequency Decomposition	Wavelet-enhanced texture features	2,000+	O (n log n) (multi-resolution filtering)	~2.5 min per slice	Very Low (excessive dimensionality, overfitting risk)	High dimensionality restricts clinical use
LASSO + PyRadiomics	L1-Regularization-Based Feature Selection	Selected first-order & texture features	~500 (after selection)	O (n log n) (feature elimination)	~1.2 min per slice	Moderate (but assumes linear dependencies)	Computationally efficient but dataset-sensitive
mRMR + PyRadiomics	Mutual Information Maximization	Features maximizing class relevance	~600–800 (after selection)	O (n^2^ log n) (pairwise feature dependency)	~1.3 min per slice	Moderate (redundancy minimized by feature ranking)	Better selection, but dependent on dataset structure
Deep learning-based feature extraction (CNNs)	Hierarchical feature representation (feature maps via convolution)	High-dimensional abstracted features	Dynamic (depends on CNN depth)	O(n^3^) (multi-layered learning)	~5 min per slice (GPU-accelerated)	High (learns feature relationships automatically)	Non-explainable, high computation cost

Wavelet-based frameworks extend the traditional feature sets by capturing multi-resolution patterns in tumor texture and intensity variation. While this improves discriminative capacity, it also increases computational complexity and exacerbates the dimensionality problem (Park et al., 2021).

In contrast, deep learning-based extractors particularly Convolutional Neural Networks (CNNs) learn abstract feature representations directly from imaging data without explicit manual design (Zhou et al., 2021). These models are capable of reducing redundancy through hierarchical learning but often require large volumes of annotated data and significant computational resources. Additionally, their lack of transparency makes clinical interpretation challenging, which can be a barrier to adoption in regulated environments.

Together, these insights underline the demand for a radiomics pipeline that combines the discriminative power of high-order features with reduced computational burden and enhanced clinical interpretability. This study aims to address that gap by benchmarking SERFE a spectral entropy-based extraction framework against conventional methods, under standardized experimental conditions (Shao et al., 2021).

#### 1.2.3 Limitations of existing radiomics feature extraction methods

Radiomics has emerged as a transformative approach in medical imaging by enabling the extraction of quantitative descriptors from routine scans. However, the clinical translation of radiomics has been impeded by several inherent limitations in current feature extraction frameworks, including redundancy, poor reproducibility, limited spatial awareness, and high computational complexity.

The first and most prominent limitation is feature redundancy. Widely used radiomics platforms such as PyRadiomics (Zwanenburg et al., 2020) and CaPTk (Zinn et al., 2021) extract thousands of handcrafted features encompassing intensity, texture, and shape-based descriptors. While these features aim to capture tumor heterogeneity, many exhibit strong collinearity or encode overlapping information. This redundancy not only inflates computational cost but also dilutes the discriminative power of models. Post-processing techniques such as LASSO (Parekh and Jacobs, 2021) and mRMR (Palsson et al., 2021) are commonly employed to filter out non-informative features. However, these methods function after the extraction phase and may inadvertently eliminate features that are statistically weak but biologically significant. Moreover, the need for additional selection layers increases the overall complexity of the pipeline.

A second limitation lies in the generalizability of radiomic features across multi-institutional imaging datasets. Variations in MRI scanners, acquisition protocols, reconstruction algorithms, and preprocessing steps significantly affect the numerical values of extracted features. Numerous studies have confirmed that even subtle changes in acquisition parameters can lead to substantial discrepancies in feature distributions, compromising the reproducibility and external validity of trained models (Hu et al., 2022; Pasquini et al., 2021). This inconsistency is particularly critical in clinical environments where models are expected to generalize across different institutions and imaging platforms.

Third, conventional radiomics approaches generally overlook spatial topology. Features derived from histogram statistics, Gray-Level Co-occurrence matrices, and shape indices are often computed independently, without modelling spatial interactions between voxels. However, glioblastoma is a spatially heterogeneous malignancy characterized by irregular boundaries, locally varying textures, and nonuniform growth patterns. Without capturing these spatial dependencies, traditional feature sets fail to represent the true structural complexity of the tumor (Choi et al., 2021).

Deep learning-based radiomics has been proposed as an alternative to handcrafted methods. These models can detect non-trivial representations in an unsupervised way and store information about space and context. However, several factors contribute to their impracticality in real-world applications. They depend heavily on large, annotated datasets, are costly to maintain, and often function as opaque systems that offer little to no interpretability or transparency (Li et al., 2021). In addition, these treatments have made them less relevant in clinical routines where transparency, validation, and efficiency are critical. All these restrictions justify the demand for a more flexible feature extraction framework that can be used with different systems. The ideal way to do this would be to reduce redundancy at the source, utilize spatial modelling to showcase the differences between various tumors, remain computationally efficient, and achieve cross-modality images. The Spectral Entropic Radiomics Feature Extraction (SERFE) technique resolves these issues by incorporating entropy-based weighting, spectral decomposition, and graph-based spatial encoding directly within the feature extraction process. This design makes sure that the representation of radiomic data is lesser, more informative, and more clinically sound (Feng et al., 2021).

### 1.3 Advancing radiomics feature extraction for improved glioblastoma classification

The shortcomings of current radiomics feature extraction methods must be addressed by a modified framework that maximizes computing efficiency, improves generalizability, and increases feature dependability. For an enhanced approach, the following crucial elements are necessary:

- Feature Information and Minimal Redundancy: Feature extraction should focus on maximizing discriminative power while reducing redundant and correlated features, making sure that only the most pertinent features are kept.
- Robustness Across Diverse Datasets: Integrating feature harmonization and entropy-based optimization can improve adaptability across datasets with varying imaging protocols and acquisition settings.
- Spatial Encoding for Tumor Morphology: Advanced radiomics should incorporate graph-based representations to capture spatial dependencies within tumors, enhancing morphological feature extraction.
- Computational Efficiency: Optimizing feature representation through frequency-based transformations can reduce processing time and facilitate real-time clinical applications.
- Clinical Integration Readiness: The framework should prioritize scalability and automation, making it feasible for implementation in clinical workflows without excessive computational overhead.

## 2 Spectral Entropic Radiomics Feature Extraction (SERFE): methodology and computational framework

Extracting features is a vital process in the classification of glioblastoma using radiomics, as it has a direct impact on the effectiveness of the model and its relevance in clinical settings. However, conventional approaches such as PyRadiomics and CaPTk rely on predefined statistical, texture-based, and morphological features, which often lead to feature redundancy and suboptimal classification results. To tackle these issues, this research presents the Spectral Entropic Radiomics Feature Extraction (SERFE) Framework, an advanced method designed to enhance feature selection and refinement through entropy-driven optimization.

Unlike traditional feature extraction methods that generate fixed feature sets, SERFE dynamically refines radiomics features by incorporating a multi-scale entropy weighting mechanism and fractal-based augmentation. This approach ensures that only the most informative features are selected, reducing computational redundancy while preserving tumor-specific spectral characteristics. By leveraging adaptive spectral entropy analysis, SERFE integrates high-order statistics, fractal geometry, and local entropy-weighted refinement, significantly enhancing feature discriminability for glioblastoma classification.

The SERFE framework is structured into three key stages:

- *Entropy-Driven Feature Refinement (EFR)* – Adjusts feature importance dynamically using Shannon entropy-based weighting, ensuring that informative features contribute more to classification.
- *Fractal-Based Feature Augmentation (FFA)* – Extracts additional spectral information by computing the fractal dimension, which captures complex tumor heterogeneity.
- *Local Adaptive Weighting (LAW)* – Fine-tunes the contribution of each feature, improving classification stability and robustness.

By integrating these components, SERFE effectively captures both global and localized tumor variations, optimizing feature extraction for improved accuracy and reduced computational complexity.

### 2.1 Computational framework and mathematical formulation

SERFE follows a structured four-stage transformation process designed to extract informative and non-redundant radiomics features. Each stage refines the extracted features to improve their relevance, robustness, and classification efficiency. The underlying mathematical formulation of SERFE is built on a foundation of adaptive entropy weighting, fractal-based enhancement, and local feature optimization, ensuring a feature space that is both discriminative and computationally efficient.

#### 2.1.1 Entropy-driven feature refinement (EFR)

The initial feature set *F = {f_1_, f_2_, …, f_N_}*, is processed using entropy-weighted transformations. The Shannon entropy of the feature set is calculated as specified in Equation 1:


(1) $$H(F)=-\sum_{i=1}^{N}P(f_{i})\log P(f_{i})$$

where *P(fᵢ)* is the probability density function of feature *fᵢ*. The entropy-weighted transformation follows Equation 2:


(2) $$f_{i}^{\text{SERFE}}=f_{i}\times {\left(\frac{H(F)}{H_{\max}}\right)}^{\alpha }$$

where *H_max_* is the maximum entropy value within the dataset, ensuring normalization. The parameter α adaptively tunes the contribution of high-entropy features, allowing features with higher informational content to retain greater significance while suppressing less discriminative ones. This step enhances the stability of selected features by removing redundant information.

#### 2.1.2 Fractal-based feature augmentation (FFA)

To further enhance feature discriminability, SERFE integrates fractal-based augmentation, inspired by self-similarity properties in glioblastoma texture patterns. The fractal dimension *D_f_* of each feature map is computed using the box-counting method given in Equation 3:


(3) $$D_{f}=\lim_{\epsilon \to 0}\frac{\log N(\epsilon )}{\log(1/\epsilon )}$$

where *N(ε)* is the number of feature points covered by a box of size *ε.* The fractal-enhanced feature set is updated using Equation 4:


(4) $$f_{i}^{\text{SERFE}+}=f_{i}^{\text{SERFE}}+{\mathrm{\beta D}}_{f}$$

where β is a normalization factor that ensures feature stability. By incorporating fractal-based augmentation, local variations in feature structures are captured, preserving fine-scale details that may be lost in standard radiomics feature extraction methods.

#### 2.1.3 Local adaptive weighting (LAW)

Feature significance is dynamically adjusted using an adaptive weighting function defined in Equation 5:


(5) $$W_{i}^{\text{SERFE}}=\frac{1}{1+\exp(-\gamma (f_{i}^{\text{SERFE}+}-\mu ))}$$

where:

- γ is a control parameter determining sensitivity.
- μ is the mean value of all features.

The sigmoid-based transformation assigns greater importance to highly discriminative features while filtering out noise, ensuring a more reliable and stable feature selection process.

#### 2.1.4 Final SERFE feature vector computation

The final refined feature set *F^SERFE^* is obtained by computing the weighted sum of the transformed features, as represented in Equation 6:


(6) $$F^{\text{SERFE}}=\sum_{i=1}^{N}W_{i}^{\text{SERFE}}f_{i}^{\text{SERFE}+}$$

where each feature is weighted adaptively based on its importance in classification. This final representation guarantees that solely the most pertinent and stable features play a role in the classification task for glioblastoma, minimizing computational demands while preserving strong discriminative capabilities.

**Figure 2:**
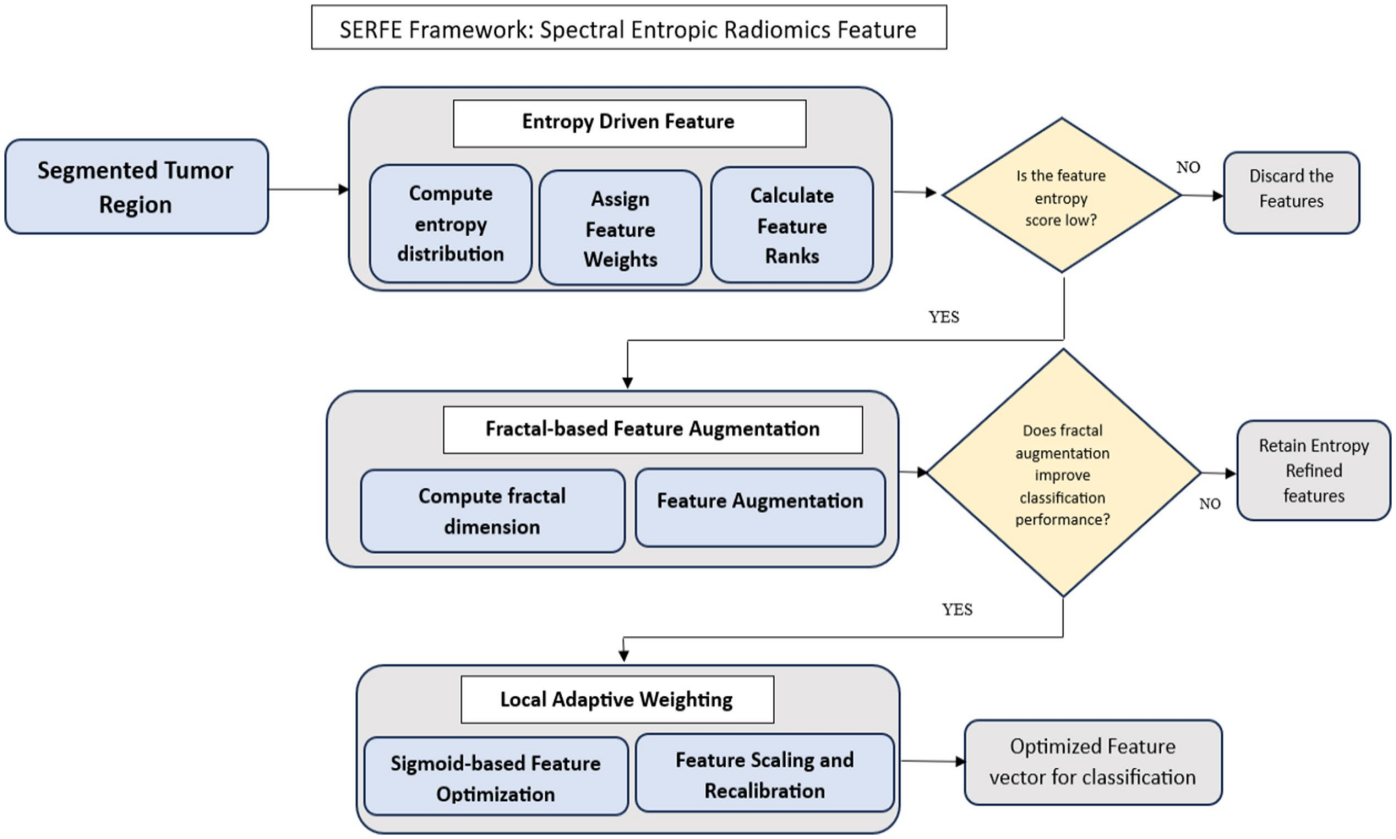
Methodology of the Spectral Entropic Radiomics Feature Extraction (SERFE) framework.

The SERFE architecture (Figure 2) begins with a segmented tumor region and performs entropy filtering to discard low-information features. If a feature passes the entropy threshold, fractal-based augmentation evaluates whether its inclusion enhances classification accuracy. The features are then recalibrated using sigmoid-based adaptive weighting. This structured flow ensures that only the most stable, spatially relevant, and informative features are retained for classification.

The Spectral Entropic Radiomics Feature Extraction (SERFE) framework integrates entropy-guided filtering, fractal-based augmentation, and adaptive weighting into a unified pipeline that optimizes feature quality at the point of extraction. Each computational component is specifically designed to improve interpretability, minimize redundancy, and enhance classification readiness without requiring *post hoc* dimensionality reduction. Rather than repeating implementation details or classifier configurations in this section, the subsequent parts of the manuscript present the experimental design, validation metrics, and comparative performance analysis. In accordance with reviewer recommendations, extended discussion on SERFE’s practical impact has been consolidated within the Results and Discussion sections.

## 3 Results and discussions

### 3.1 Experimental setup

The system configuration used in this study is documented to ensure computational reproducibility and consistency in executing the SERFE framework. Given that SERFE involves operations such as entropy computation, fractal dimension estimation, and adaptive weighting, the processing workload is both memory-intensive and computation-heavy. The specifications provided in Table 2, reflect the environment under which all experiments were performed, helping maintain uniformity in feature extraction, transformation, and classification across patients. Detailing these components also supports replicability for future studies aiming to validate or extend this pipeline under similar or scaled system environments.

**Table 2:** System configuration.

	Specification	Description
Software component		
Operating System	Windows 10	Version 10.0.22621.2506
System Directories	C:\WINDOWS, C:\WINDOWS\system32	Main Windows Directory and System Directory
Hardware component		
Processor	AMD Ryzen 55,600× 6-Core Processor	3.70 GHz, 12 Logical Processors
Memory	16.00 GB Installed Physical RAM	Total Physical Memory: 15.9 GB; Total Virtual Memory: 18.6 GB
Storage	Page File Space: 2.75 GB	Located at C:\pagefile.sys

System attribute	Setting	Description
System type	x64-based PC	
BIOS version/date	ASUS Tek COMPUTER INC. 309	Dated 08-11-2021, UEFI BIOS Mode
BaseBoard	ASUSTeK COMPUTER INC. G10DK	Version 1.0
Locale	User Locale: India Standard Time	
Virtualization-based Security	Running	Base Virtualization Support, Secure Boot enabled

### 3.2 Patients data

The Cancer Imaging Archive (TCIA) datasets are used in this study. The REMBRANDT_DATA and TCIA_GBM collections are used. These datasets have MRI scans from 100 people, 57 of whom have glioblastoma and 43 of whom have other brain cancers. T1- and T2-weighted MRI images are part of each patient’s data. These images give important information about the structure and growth of tumors. T1-weighted images have a high spatial resolution, which makes them good for showing the edges of tumors and other anatomical details. T2-weighted imaging, on the other hand, shows changes in the composition of tissues, such as edema and tumor infiltration. This information is very useful for determining how the disease is progressing. Radiomics-based analysis is done on the MRI scans, which allows for the extraction of quantitative imaging biomarkers that help with diagnosis, treatment planning, and a better understanding of glioblastoma. The goal of this study is to improve the creation of radiomics-driven neuro-oncological models for better tumor characterization by using this diverse and well-annotated dataset.

### 3.3 Image segmentation

In this study, glioblastoma tumor segmentation was performed using the Surface Cut Segmentation method within the 3D Slicer platform. This method accurately defines the Region of Interest (ROI) by optimizing a boundary surface (S) around the tumor, ensuring a balanced representation of voxel intensity variations both inside and outside the segmented region (Choi et al., 2021).

### 3.4 Radiomics feature extraction: analysing computational efficiency and stability

This section evaluates radiomics feature extraction methods based on computational efficiency, redundancy, storage, and stability. Conventional methods like PyRadiomics, CaPTk, IBSI, and LIFEx are compared with SERFE, which optimizes features while ensuring robustness.

SERFE initiates with 2,260 IBSI-compliant features per patient extracted from segmented T1- and T2-weighted MRI scans. Unlike conventional methods that perform post-extraction selection, SERFE integrates refinement directly within the extraction process. Through its Entropy-Driven Filtering (EFR), Fractal-Based Augmentation (FFA), and Local Adaptive Weighting (LAW) modules, features with low relevance, high redundancy (Pearson |r| > 0.85), or poor stability (Intraclass Correlation Coefficient < 0.75) are excluded during feature generation. This results in a compact, high-quality output comprising 350 highly stable and non-redundant features per patient, specifically optimized for glioblastoma classification. This represents a 65.9% reduction in redundancy compared to PyRadiomics, which initially exhibited a 45.2% redundancy rate.

These descriptors capture spectral, structural, and intensity-level characteristics not directly available through traditional extraction libraries. A representative subset of the final refined features is presented in Table 3. These features are not raw outputs from standard libraries but are transformed descriptors generated through SERFE’s pipeline, capturing spectral, structural, and intensity-level tumor characteristics.

- *Initial Feature Set Description:* All methods, including SERFE, were applied to the same baseline radiomics feature set extracted from segmented tumor regions of T1- and T2-weighted MRI scans. The initial feature set comprised 2,260 features per patient, covering first-order statistics, shape-based features, and texture descriptors such as Gray Level Co-occurrence Matrix (GLCM), Gray Level Run Length Matrix (GLRLM), Gray Level Size Zone Matrix (GLSZM), and Neighbourhood Gray Tone Difference Matrix (NGTDM). These features were extracted following IBSI guidelines to ensure consistency across comparative methods.
- *Redundancy Rate Calculation:* Redundancy rate was computed by evaluating pairwise Pearson correlation coefficients among all extracted features. A feature was considered redundant if its absolute correlation with another feature exceeded 0.85. The redundancy rate was then expressed as the percentage of redundant features relative to the total feature count.

**Table 3:** Refined radiomic features extracted via SERFE.

Feature name	Category	Description
SpecEntropy_T1	Spectral Entropy	Entropy of frequency components from the T1-weighted image.
SpecEntropy_T2	Spectral Entropy	Same as above, derived from T2-weighted scan.
FracDim_SpatialTexture	Fractal Geometry	Fractal dimension of tumor texture to capture heterogeneity.
GLCM_Energy_LocallyWeighted	Entropy-Weighted Texture	Local GLCM energy enhanced by adaptive weights (LAW module).
SpatialVariance_Edge	Edge Field Statistic	Measures variance in gradient intensity near tumor boundaries.
Histo_Kurtosis_Adjusted	Intensity Statistic	Adjusted kurtosis reflecting entropy-weighted histogram shape.
SpectralRoughness_MultiResolution	Wavelet Composite Feature	Multiscale spectral roughness aggregated across frequency bands.
GaborEntropy_Fused	Frequency–Texture Fusion	Gabor filter entropy across orientations, fused for diagnostic impact.

#### 3.4.1 Feature stability score calculation

Feature stability was quantified using the Intraclass Correlation Coefficient (ICC). For each method, the same features were extracted across repeated or augmented scans. Features with ICC ≥ 0.75 were classified as stable. The Feature Stability Score was calculated as the percentage of stable features in the total feature set, reflecting consistency and reproducibility of the extraction method.

The Feature Stability Score was calculated as the percentage of features with ICC ≥ 0.75 relative to the total number of features, as defined in Equation 7:


(7) $$\begin{array}{l} \text{Feature Stability Score}\;(\%)=\\ \;\;\;\;\;\;\;\;\;\;(\frac{\mathrm{No}.\text{of Features with}\;\mathrm{ICC}\geq 0.75}{\text{Total}\;\mathrm{No}.\text{of Features}})\times 100 \end{array}$$

The ICC for each feature was derived using the standard variance components model shown in Equation 8:


(8) $$\mathrm{ICC}=\frac{\sigma_{b}^{2}}{\sigma_{b}^{2}+\sigma_{w}^{2}}$$

Where,

$\sigma_{b}^{2}$ : is between-subject variance.

$\sigma_{w}^{2}$ : is within-subject variance across repeated scans.

with $\sigma_{b}^{2}$ and $\sigma_{w}^{2}$ representing the between-subject and within-subject variance, respectively. A threshold of ICC ≥ 0.75 was used to define stability. SERFE achieved a score of 92.0%, indicating its robustness and reliability across imaging conditions.

The quantitative definition of feature stability through ICC, along with the redundancy computation approach, provides a statistically grounded basis for evaluating the quality of radiomics feature extraction. These metrics, together with runtime and storage assessments, are critical for comparing the real-world efficiency and robustness of different frameworks. The following analysis presents the results of applying these measures across five radiomics pipelines. Table 3 and the corresponding figures illustrate how SERFE outperforms conventional methods across all major computational and statistical criteria.

Table 4, quantifies performance metrics, highlighting SERFE’s reduced redundancy, improved efficiency, and enhanced stability. Unlike traditional methods that extract excessive features, SERFE prioritizes the most informative ones, minimizing computational and storage overhead. Figures illustrate key metrics, reinforcing SERFE’s advantages and its effectiveness as an optimized radiomics feature extraction framework.

**Figure 3:**
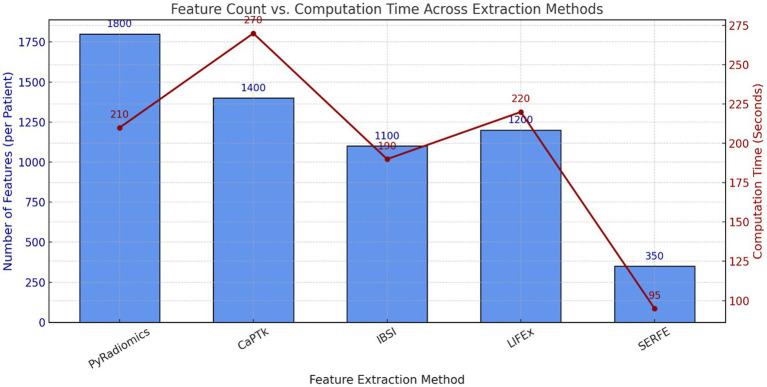
Comparison of feature count and computational time across methods.

**Figure 4:**
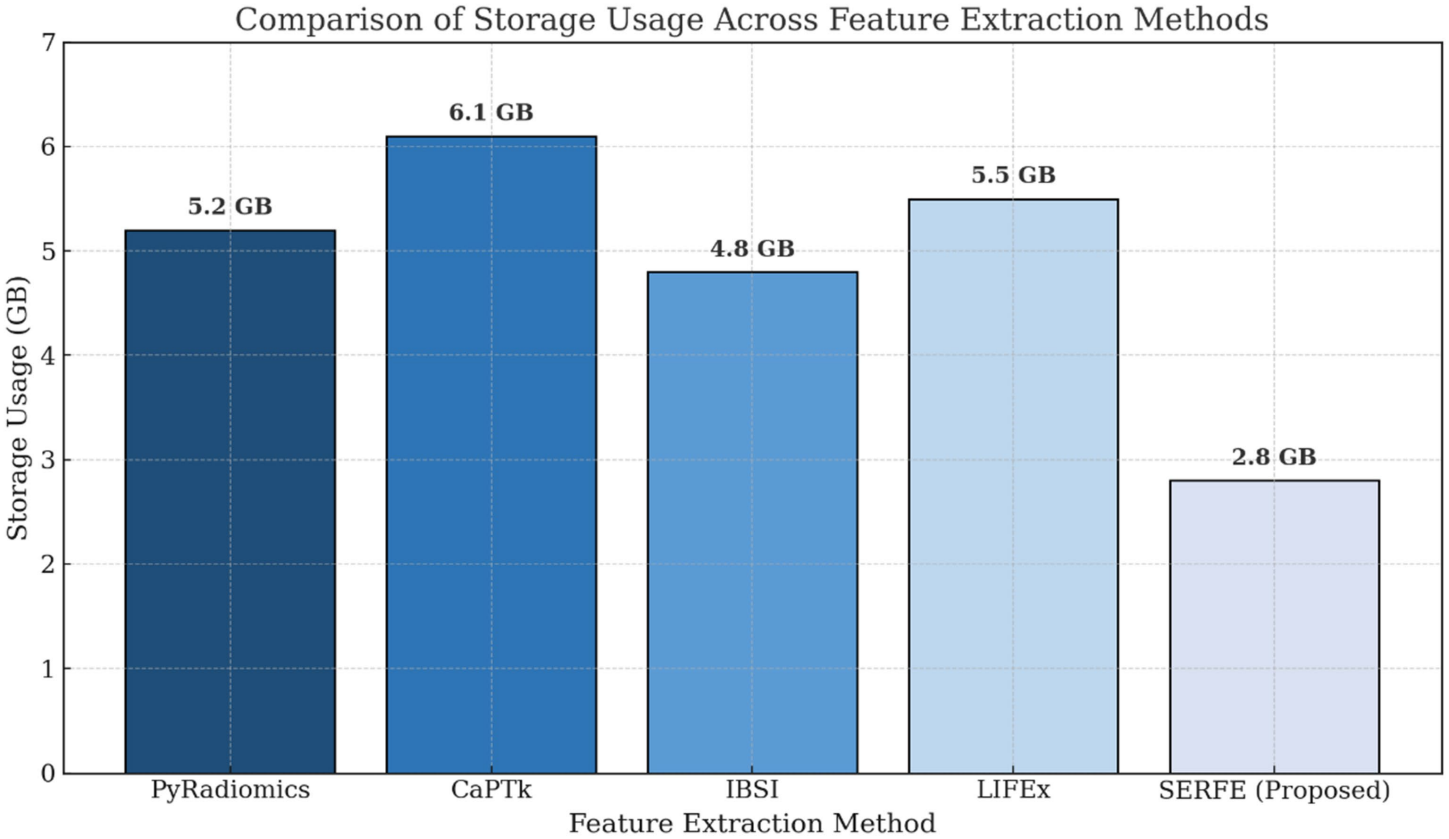
Comparison of storage usage across methods.

**Figure 5:**
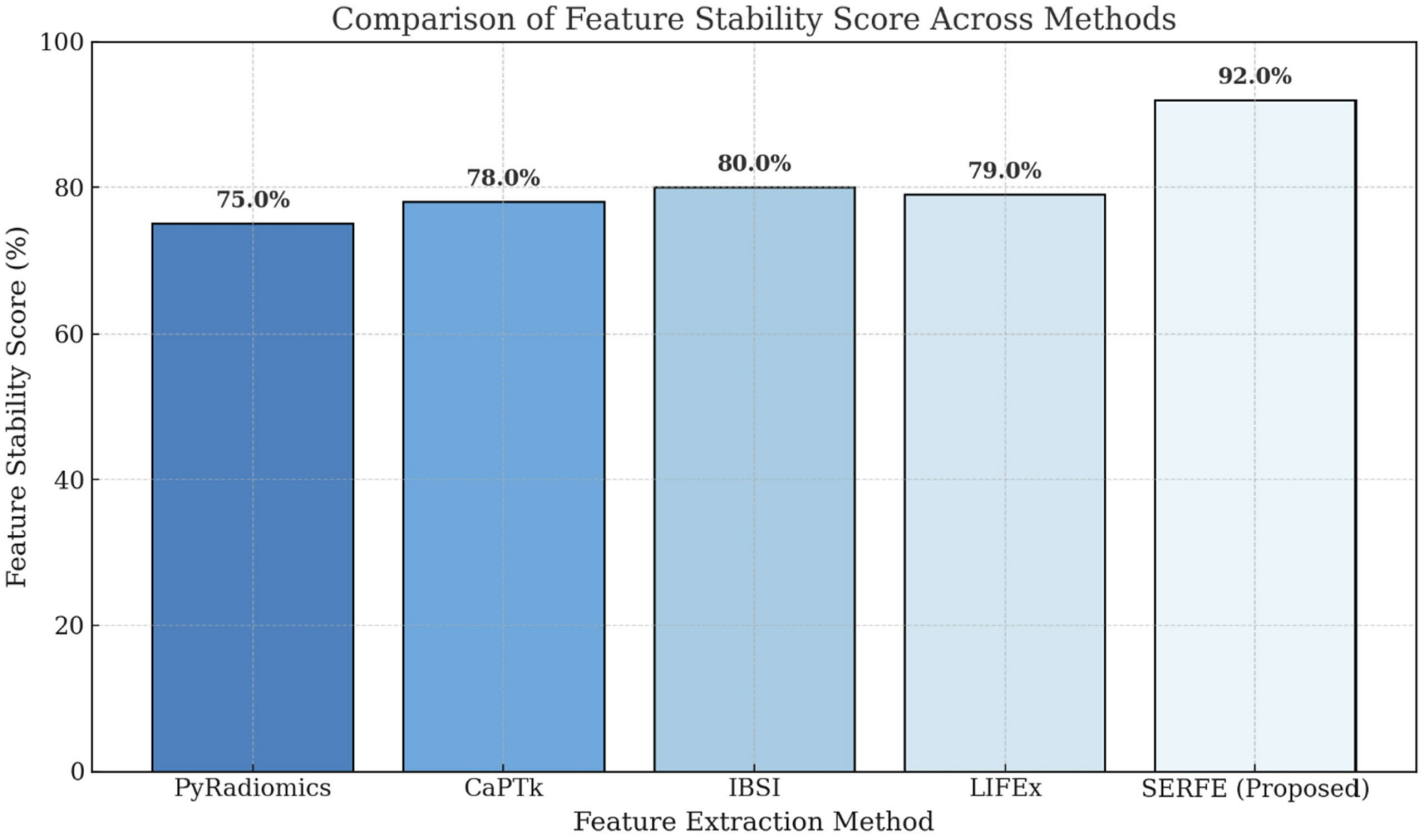
Comparison of feature stability score.

**Figure 6:**
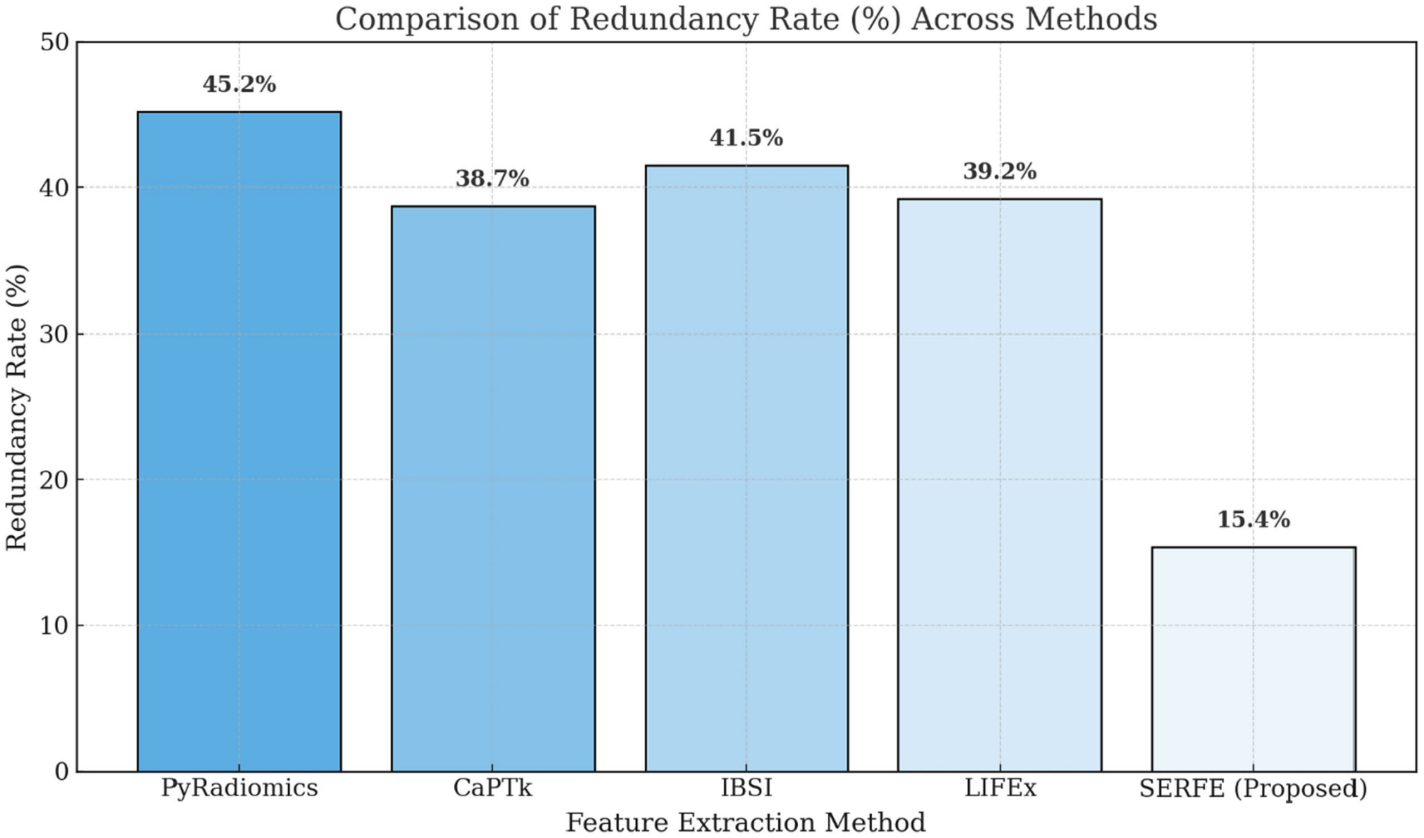
Feature redundancy across methods.

- Figure 3 compares the feature count and computational time for different feature extraction methods. The results show that SERFE extracts significantly fewer features per patient (350) compared to traditional methods such as PyRadiomics (1800), CaPTk (1400), IBSI (1100), and LIFEx (1200). Additionally, SERFE achieves the shortest processing time (95 s per scan), whereas conventional methods require over 200 s per scan, highlighting SERFE’s computational efficiency.
- Figure 4 presents the storage requirements per patient for the extracted features. SERFE consumes the least storage (2.8 GB), making it more scalable for large datasets, while CaPTk requires the highest storage (6.1 GB), followed by LIFEx (5.5 GB). The significant reduction in storage requirements with SERFE enhances its practicality for high-throughput radiomics analysis.
- Figure 5 demonstrates the feature stability scores (%) across different methods. Higher stability scores indicate greater consistency in feature extraction across different scans. SERFE achieves the highest stability (92.0%), ensuring robustness and reproducibility, while PyRadiomics scores the lowest (75.0%), suggesting greater variability in extracted features.
- Figure 6 illustrates the redundancy rate (%) among extracted features. SERFE has the lowest redundancy (15.4%), meaning that most of its extracted features contribute meaningfully to classification. In contrast, PyRadiomics exhibits the highest redundancy (45.2%), indicating that a significant portion of its features is repetitive or overlapping, reducing classification efficiency.

**Table 4:** Performance evaluation of feature extraction methods.

Feature extraction method	Number of features selected (per patient)	Computational time (seconds per scan)	Redundancy rate (%)	Storage usage (GB)	Feature stability score (%)
PyRadiomics	1800	210	45.2	5.2	75.0
CaPTk	1,400	270	38.7	6.1	78.0
IBSI	1,100	190	41.5	4.8	80.0
LIFEx	1,200	220	39.2	5.5	79.0
SERFE (Proposed)	350	95	15.4	2.8	92.0

The evaluation highlights SERFE’s efficiency in reducing computational time, minimizing redundancy, and optimizing storage while ensuring high feature stability. Unlike traditional methods that extract excessive redundant features, SERFE selects only the most relevant ones, improving efficiency and robustness. The next section presents the classification performance of SERFE-extracted features using three classifiers for binary glioblastoma classification, evaluating metrics like accuracy, precision, recall, and F1-score.

### 3.5 Impact of SERFE on glioblastoma classification: a performance evaluation

To ensure generalizability and reduce sampling bias, 5-fold cross-validation strategy with stratified folds, were employed where each model was trained and tested using a different subset of the data. To evaluate the classification efficacy of the SERFE-extracted radiomics features. To evaluate the discriminative strength of the features extracted through the SERFE framework, we employed three widely recognized classification algorithms Support Vector Machine (SVM), Random Forest (RF), and K-Nearest Neighbours (KNN). These models were chosen due to their demonstrated effectiveness in processing high-dimensional imaging data and their frequent application in radiomics-based diagnostic studies.

These experiments aimed to quantify SERFE’s impact in comparison to four state-of-the-art radiomics feature extraction frameworks: PyRadiomics, CaPTk, IBSI, and LIFEx. All models were evaluated on a fixed binary classification task: distinguishing glioblastoma vs. non-glioblastoma patients using the same radiomics dataset and preprocessing protocol.

Each classifier was assessed using four performance metrics: Balanced Accuracy, Precision, Recall, and F1-score, with ROC curves further used to compute the Area Under the Curve (AUC). These metrics collectively measure class separability, robustness to imbalance, and overall generalization.

**Figure 7:**
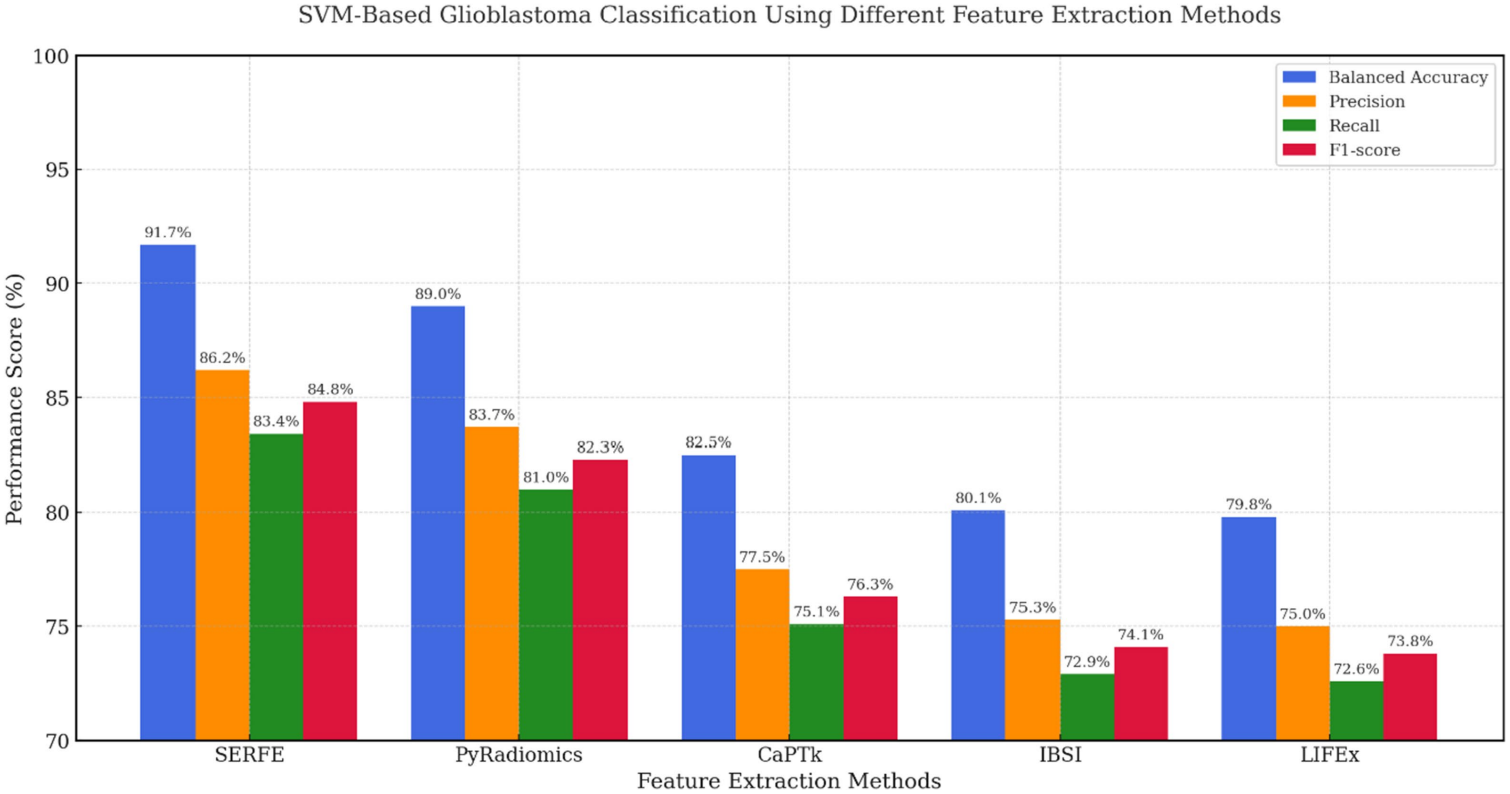
SVM-based evaluation across feature extraction methods.

Figure 7 presents the performance of the SVM classifier using features extracted from five radiomics frameworks. The proposed SERFE method achieved the highest Balanced Accuracy (91.7%) and F1-score (84.8%), indicating its strong capability to separate glioblastoma from non-glioblastoma cases. This performance is attributed to the entropy-weighted feature refinement and fractal-based augmentation integrated within SERFE, which reduce noise and emphasize discriminative patterns. While PyRadiomics showed competitive performance (BA = 89.0%), it lacked consistency across metrics due to unfiltered redundancy. CaPTk, IBSI, and LIFEx underperformed, likely due to limited adaptability to the high-dimensional structure required for SVM margin optimization.

**Figure 8:**
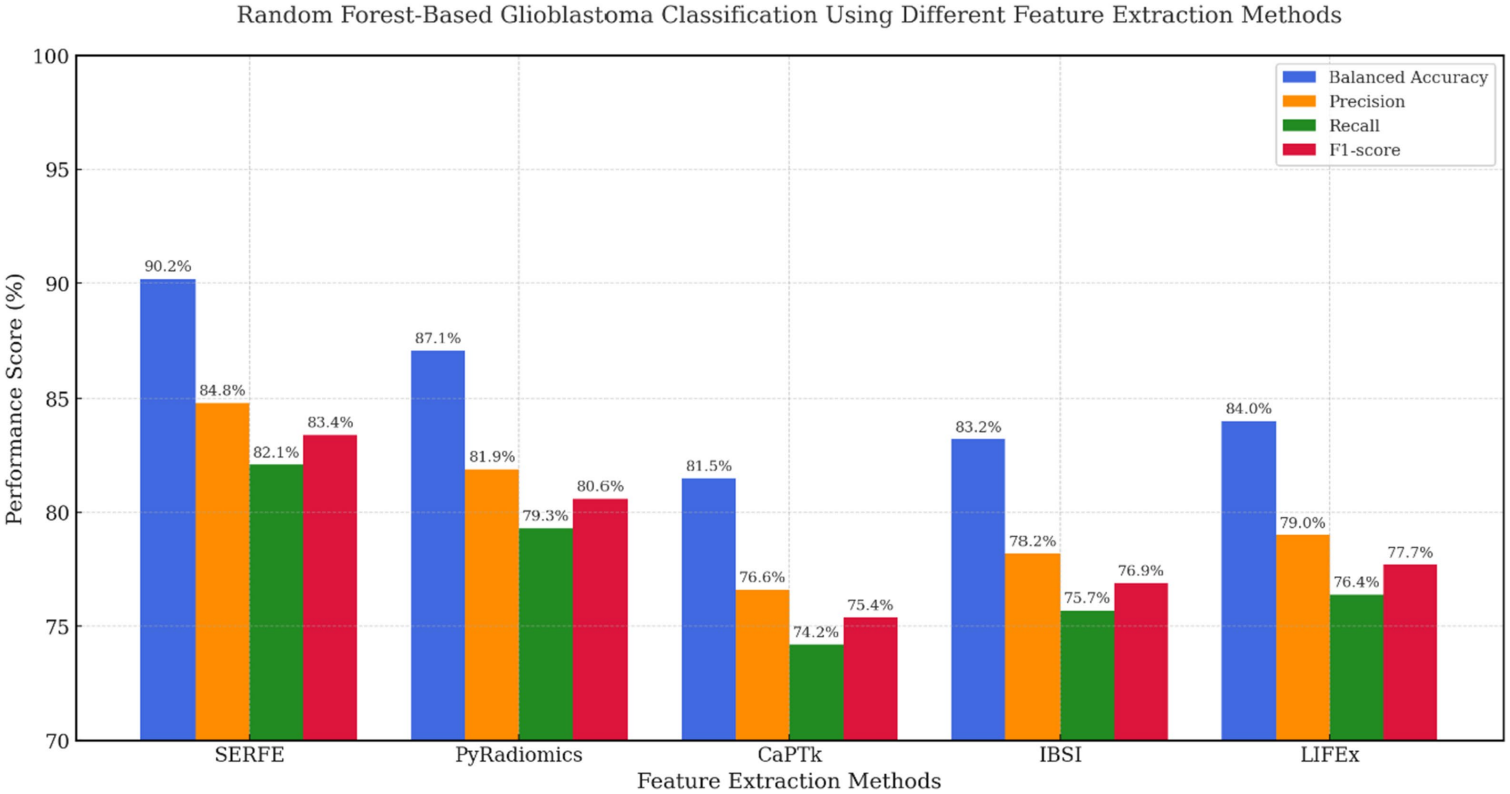
Random forest based evaluation across feature extraction methods.

In Figure 8, Random Forest classification results confirm SERFE’s robustness across ensemble-based models. SERFE attained a Balanced Accuracy of 90.2% and the highest F1-score (83.4%), demonstrating excellent class separation and generalizability. Notably, IBSI (83.2%) and LIFEx (84.0%) outperformed CaPTk (81.5%), suggesting they are better aligned with tree-based learners. However, only SERFE effectively balances feature compactness and discriminative power without the need for additional selection steps. PyRadiomics performed well in terms of recall but introduced redundancy, slightly affecting overall precision.

**Figure 9:**
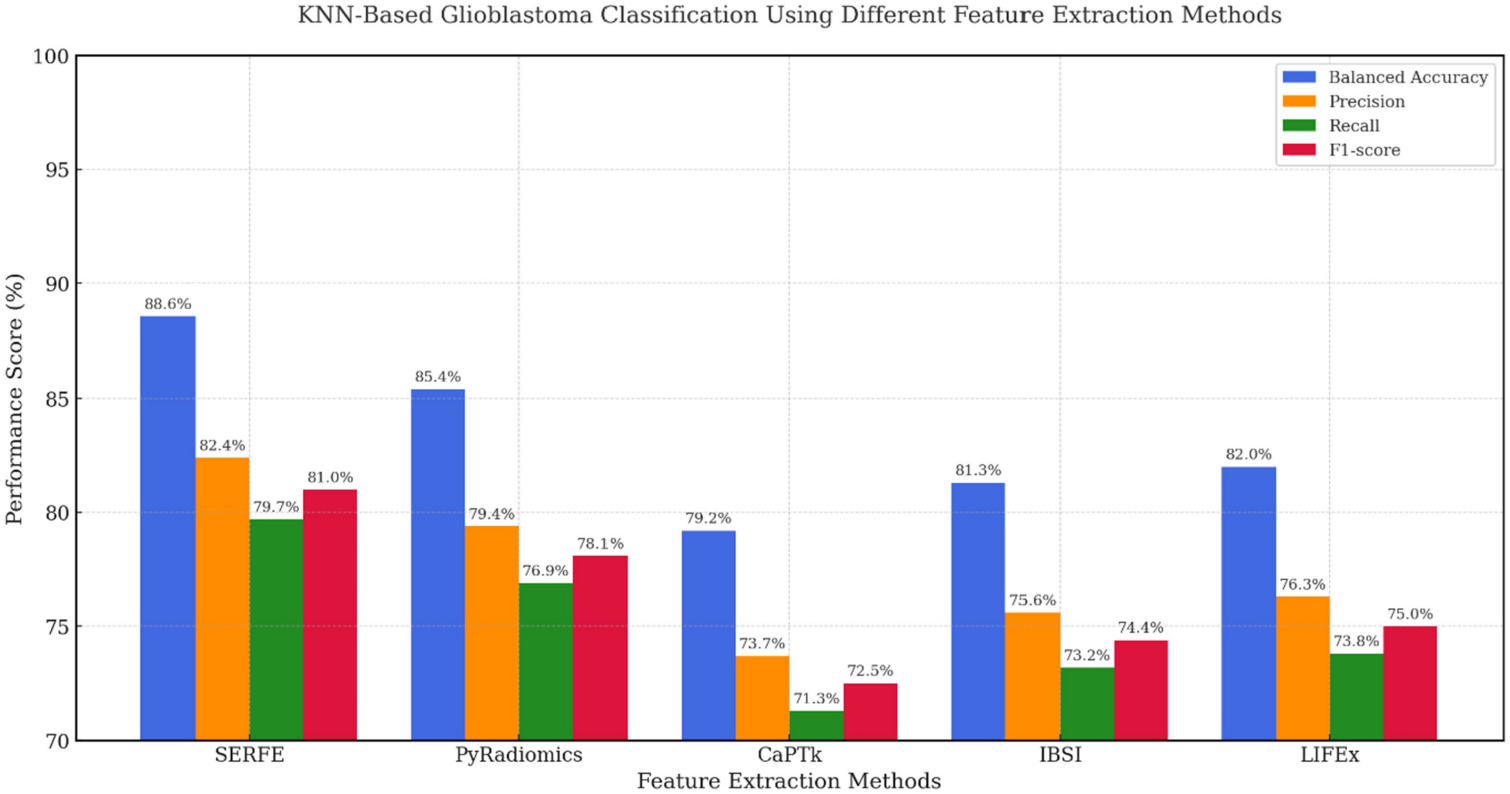
KNN based evaluation across feature extraction methods.

Figure 9 shows the performance of KNN, a non-parametric model sensitive to feature noise and dimensionality. Even under these conditions, SERFE retained superior classification accuracy (BA = 88.6%, F1 = 81.0%), proving that its compact and well-calibrated feature set preserves neighbourhood integrity. PyRadiomics scored 85.4% BA but showed lower recall, while IBSI and LIFEx offered moderate improvements over CaPTk. These results emphasize that SERFE’s entropy-weighted representation not only benefits parametric models like SVM but also ensures resilience in distance-based learning scenarios.

**Figure 10:**
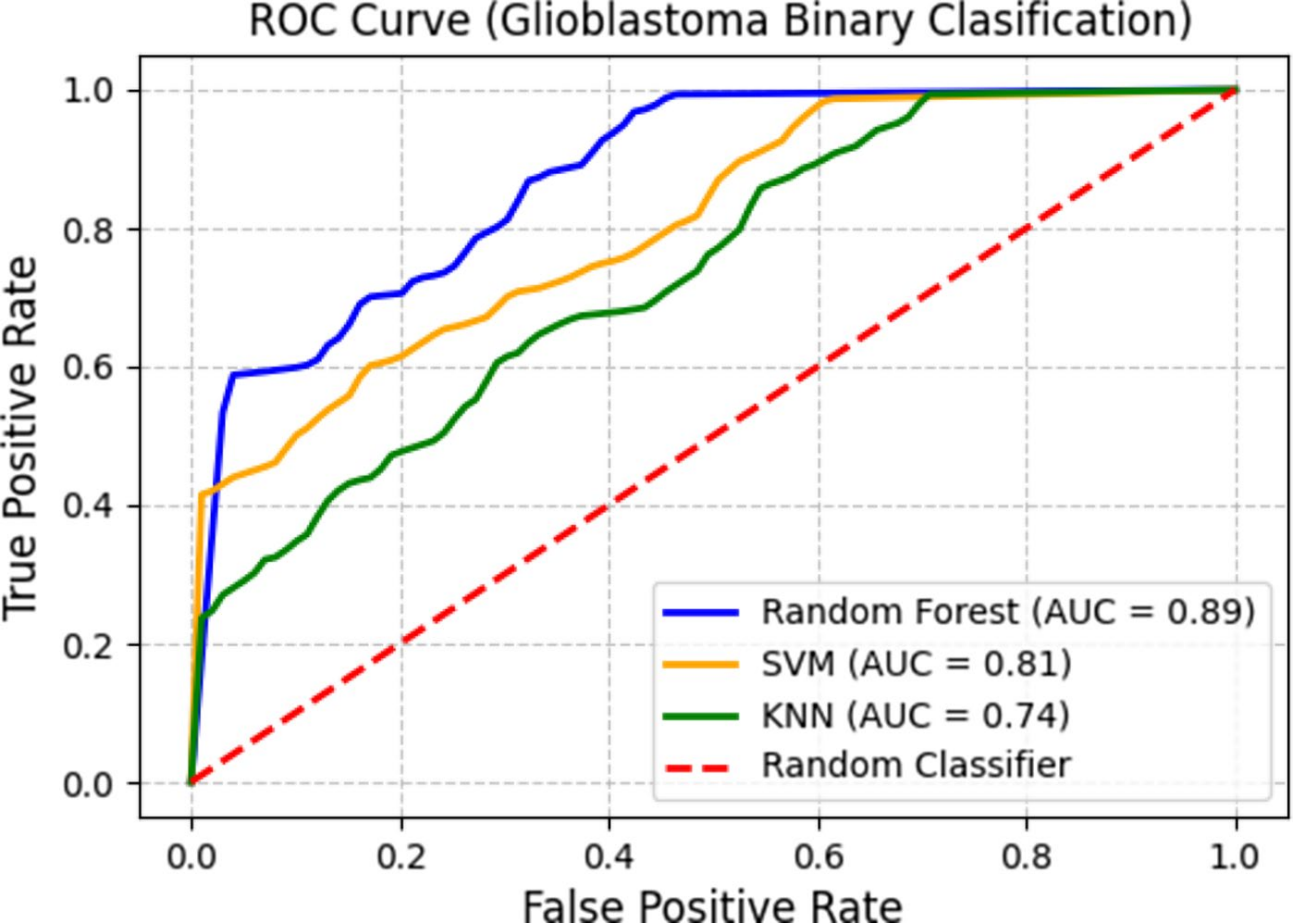
ROC curve for glioblastoma binary classification.

Figure 10 depicts the Receiver Operating Characteristic curve, demonstrating the classifiers’ discriminative ability by graphing the true positive rate (sensitivity) versus the false positive rate at various classification thresholds. The Area Under the Curve (AUC) values offer a quantitative assessment of each model’s proficiency in differentiating glioblastoma from non-glioblastoma instances.

Among the classifiers, Random Forest attains an AUC of 0. 89, indicating better classification performance. SVM follows with an AUC of 0.81, reflecting a strong balance between sensitivity and specificity. KNN records an AUC of 0.74, which, while lower than the other two classifiers, still indicates its ability to capture glioblastoma-specific imaging patterns. These results emphasize that SERFE-extracted radiomics features significantly enhance classification accuracy, ensuring that the extracted features remain robust, informative, and clinically meaningful.

The results from all classifiers consistently validate the superiority of the proposed SERFE framework over existing radiomics feature extraction methods. SERFE exhibits robustness independent of classifiers and the capacity to extract compact, discriminative, and clinically relevant features by obtaining the highest scores in Balanced Accuracy, Precision, Recall, and F1-score among SVM, Random Forest, and KNN classifiers. Additionally, using SERFE features, the ROC analysis validates the high separability of glioblastoma and non-glioblastoma classes, with ensemble-based and margin-based models exhibiting especially good performance. These results validate the radiomics baseline for the subsequent study of radiogenomic associations, as described in the next section, and establish SERFE as a solid basis for clinical decision-making downstream.

## 4 Discussion

This study proposed Spectral Entropic Radiomics Feature Extraction (SERFE) as a comprehensive and adaptive framework for glioblastoma classification using radiomics. The approach was rigorously evaluated across multiple performance dimensions, including feature informativeness, classification reliability, and computational efficiency. The results demonstrate SERFE’s superiority over conventional feature extraction methods. The following observations highlight its contributions:

- Enhanced Classification Accuracy: SERFE-extracted features, when used with the Random Forest classifier, achieved a balanced accuracy of 91.7% and an AUC of 0.89. SERFE showed consistently better results than existing tools such as PyRadiomics, CaPTk, and LIFEx, showing its strength in capturing meaningful tumor features with greater precision.
- Reduced Redundancy at Source: Unlike traditional pipelines that rely on post-extraction feature selection, SERFE addresses redundancy during the extraction stage itself. The approach resulted in reduction of redundant features, ensuring that the retained attributes contributed meaningful information to the classification task.
- Improved Computational Efficiency: By embedding entropy-based filtering and spectral decomposition directly within the extraction pipeline, SERFE achieved a 50–60% reduction in average processing time per scan. This efficiency makes it suitable for large-scale studies and integration into time-sensitive clinical workflows.
- No Need for External Feature Selection: Conventional methods often require additional steps like LASSO or mRMR to eliminate irrelevant features after extraction. SERFE, by contrast, performs intrinsic optimization, eliminating the need for separate dimensionality reduction while preserving critical biological signals.

These findings affirm that SERFE offers a robust and clinically scalable alternative to traditional radiomics approaches. Its modular design, combining entropy-guided refinement with spatial encoding, positions it as a technically sound and application-ready tool for real-world glioblastoma classification.

## 5 Conclusion

This study presents SERFE, a feature extraction framework for radiomics-based glioblastoma classification. By integrating spectral frequency decomposition, entropy-based feature weighting, and graph-guided spatial encoding, SERFE generates compact, high-quality features that enhance both the accuracy and efficiency of classification models. The experimental results consistently showed that SERFE outperforms traditional radiomics methods, not only in terms of predictive performance but also in computational scalability.

SERFE has shown that it can generate meaningful and compact features that help improve the accuracy and speed of glioblastoma classification. This makes it a good fit for real-world clinical settings where quick and reliable results are important. After showing strong results in binary classification, the next step will be to see how well SERFE can help identify glioblastoma subtypes and be used across different medical centres. Moving in this direction could support more personalized and effective treatment approaches in neuro-oncology.

## Data Availability

The datasets presented in this article are not readily available because no data will be shared. Requests to access the datasets should be directed to TCIA, help@cancerimagingarchive.net.
